# Preclinical evaluation of marketed sodium channel blockers in a rat model of myotonia discloses promising antimyotonic drugs

**DOI:** 10.1016/j.expneurol.2014.02.023

**Published:** 2014-05

**Authors:** Jean-François Desaphy, Roberta Carbonara, Teresa Costanza, Diana Conte Camerino

**Affiliations:** Section of Pharmacology, Department of Pharmacy & Drug Sciences, University of Bari-Aldo Moro, Bari I-70125, Italy

**Keywords:** Non-dystrophic myotonia, Sodium channel blockers, Mexiletine, Rat model, Patch-clamp, Over-excitability

## Abstract

Although the sodium channel blocker mexiletine is considered the first-line drug in myotonia, some patients experiment adverse effects, while others do not gain any benefit. Other antimyotonic drugs are thus needed to offer mexiletine alternatives. In the present study, we used a previously-validated rat model of myotonia congenita to compare six marketed sodium channel blockers to mexiletine. Myotonia was induced in the rat by injection of anthracen-9-carboxylic acid, a muscle chloride channel blocker. The drugs were given orally and myotonia was evaluated by measuring the time of righting reflex. The drugs were also tested on sodium currents recorded in a cell line transfected with the human skeletal muscle sodium channel hNav1.4 using patch-clamp technique. In vivo, carbamazepine and propafenone showed antimyotonic activity at doses similar to mexiletine (ED_50_ close to 5 mg/kg); flecainide and orphenadrine showed greater potency (ED_50_ near 1 mg/kg); lubeluzole and riluzole were the more potent (ED_50_ near 0.1 mg/kg). The antimyotonic activity of drugs in vivo was linearly correlated with their potency in blocking hNav1.4 channels in vitro. Deviation was observed for propafenone and carbamazepine, likely due to pharmacokinetics and multiple targets. The comparison of the antimyotonic dose calculated in rats with the current clinical dose in humans strongly suggests that all the tested drugs may be used safely for the treatment of human myotonia. Considering the limits of mexiletine tolerability and the occurrence of non-responders, this study proposes an arsenal of alternative drugs, which may prove useful to increase the quality of life of individuals suffering from non-dystrophic myotonia. Further clinical trials are warranted to confirm these results.

## Highlights

• Seven sodium channel blockers show antimyotonic activity in a rat model of myotonia.
• The ED_50_ value ranges from 0.1 (riluzole, lubeluzole) to 5 mg/kg (mexiletine, carbamazepine).
• The drugs use-dependently block hNav1.4 channels in cells in myotonic-like conditions.
• The IC_50_ values in vitro were well linearly correlated with the ED_50_ values in vivo.
• The study discloses promising new therapeutic options for myotonic patients.

## Introduction

Myotonic disorders are characterized by skeletal muscle stiffness after voluntary contraction or percussion. Inherited myotonias include dystrophic and non-dystrophic diseases and can be subdivided in various disease entities on the basis of clinical phenotype and genotype (Matthews et al., 2010; Rayan and Hanna, 2010; Trivedi et al., 2013b). Non-dystrophic myotonias (NDM) are caused by mutations in genes encoding the voltage-dependent chloride channel (*CLCN1* gene, ClC-1 protein) or sodium channel (*SCN4A* gene, Nav1.4 protein), which are expressed exclusively in skeletal muscles. The reduced activity of mutated ClC-1 channels or increased activity of mutated Nav1.4 channels determine a pathological sarcolemma hyperexcitability with occurrence of high-frequency action potential discharges; the consequent difficulty in muscle relaxation is responsible for the characteristic stiffness of myotonic muscle. NDM can be chronically debilitating due to pain and muscle stiffness, which are associated with frequent falls and disability. In the more severe cases, myotonia can prohibit sport practice, limit ability at school, and alter physical growth.

Pharmacotherapy is often needed for myotonic patients. Today, the class IB antiarrhythmic, mexiletine, is considered as the drug of choice. By blocking skeletal muscle sodium channels, mexiletine can contrast high frequency firing of myotonic action potentials. Recent clinical trials have confirmed the usefulness of mexiletine in both dystrophic and non-dystrophic myotonias, irrespective of the culprit gene (Logigian et al., 2010; Statland et al., 2012). Consequently, mexiletine was recently appointed by FDA and EMA as an orphan drug in NDM. Mexiletine preferentially binds the open and/or inactivated sodium channels, resulting in a use-dependent block that is thought to constitute the basis of the selective action of mexiletine on pathologic hyperactive tissues (Conte Camerino et al., 2007; Desaphy et al., 1999; Wang et al., 2004).

Nevertheless not all the myotonic individuals obtain benefits from mexiletine, because the drug can induce side effects limiting patient compliance (mainly epigastric discomfort, nausea, tremor, anxiety, dizziness, lightheadedness, and headaches). Particular attention must be also paid to cardiomyopathic patients, who may be exposed to life-threatening complications. In addition, non responders to mexiletine therapy have been occasionally observed, likely due to pharmacogenetic mechanisms (Desaphy et al., 2001, 2013a, 2013b, 2013c). Last but not least, mexiletine was withdrawn from the market in several countries, leaving patients and doctors with an unmet medical need. It is thus likely that a number of myotonic individuals would obtain significant improvement of quality of life from the individuation of new efficient and safe antimyotonic drugs.

Other sodium channel blockers have been anecdotally reported to exert antimyotonic activity, including carbamazepine, flecainide, and propafenone, although no randomized clinical trial has been performed in NDM (Alfonsi et al., 2007; Desaphy et al., 2013a; Lyons et al., 2010; Rosenfeld et al., 1997; Savitha et al., 2006; Sechi et al., 1983). Flecainide and propafenone resulted efficient in patients with specific sodium channel mutations, who were resistant to mexiletine (Alfonsi et al., 2007; Desaphy et al., 2013a; Rosenfeld et al., 1997). Nevertheless, there is no available information regarding the relative efficiency of these drugs in the whole myotonic population. It is worth to note that no chloride channel openers are available.

Starting from these considerations, we have recently developed a preclinical rat model of myotonia in vivo to allow drug screening (Desaphy et al., 2013b). In this model, inhibition of the ClC-1 chloride channel by anthracen-9-carboxylic acid (9AC) induces muscle stiffness that increases the time of righting reflex (TRR) of the rat. Thus we were able to quantitatively assess the in vivo antimyotonic activity of orally-administrated mexiletine and β-adrenergic drugs that also block sodium channels in a use-dependent manner (Desaphy et al., 2003, 2013b).

In the present study, we used this preclinical model to evaluate in vivo the antimyotonic activity of a number of marketed sodium channel blockers. The tested drugs were chosen because they were previously reported to relieve myotonia in humans (mexiletine, carbamazepine, flecainide, and propafenone) (Alfonsi et al., 2007; Desaphy et al., 2013a; Lyons et al., 2010; Rosenfeld et al., 1997; Savitha et al., 2006; Sechi et al., 1983), and/or to exert significant block of human skeletal muscle sodium channels in cell lines (orphenadrine, lubeluzole, and riluzole) (Desaphy et al., 2001, 2004, 2009, 2012, 2013c). Patch clamp experiments were performed to verify the correlation between use-dependent block of heterologously-expressed hNav1.4 channels and antimyotonic efficiency in vivo.

## Materials and methods

### Animal care and in vivo experiments

The experiments were performed in accordance with the Italian Guidelines for the use of laboratory animals, which conforms to the European Union Directive for the protection of experimental animals (2011/63/EU), and received approval from the Animal Experimentation Ethic Committee of the University of Bari (CESA prot. 7/12) and Italian Health Department (Decreto n. 91/2013-B). All efforts were made to minimize animal suffering and to reduce the number of animals used.

Adult WISTAR rats (350–500 g) purchased from Charles River—Italy were housed individually and given food and water ad libitum. In a typical daily experiment, three/four rats were randomly assigned to receive an intraperitoneal injection of 30 mg/kg anthracen-9-carboxylic acid (9AC), as previously described (Desaphy et al., 2013b). Ten minutes after 9-AC injection, the animals were given drug or vehicle per os using an esophageal cannula. Myotonia state was assayed by measuring the time of righting reflex (TRR), that is the time taken by the rat to turn back on his four limbs after having been positioned in supine position. Ambient temperature was maintained to 20–22 °C. The TRR was determined 10 min before and 10, 30, 60, 120, and 180 min after 9AC administration. At each time point, the TRR was calculated as the average of 7 trials, which allows obtaining a S.E.M. minor than 10% of the mean. A 1-minute interval was respected between two trials to avoid any warm-up phenomenon. Typically, a control rat takes less than 0.5 s to turn back on his limbs, whereas, 30 min after 9AC injection, a myotonic rat shows a TRR greater than 3 s. An antimyotonic drug is expected to reduce the TRR with respect to vehicle. Effect of 9AC was maximal 30 min after injection and reversed spontaneously within 15 h. At the end of each experimental day, the tested rats were allowed a resting period of at least 48 h before to reintegrate the reserve pool. A number of experiments have been performed blindly, meaning that the two operators who measured the TRR in the four rats in the daily experiment were not aware of the drug dose given to each rat. No apparent difference was found between blind and open experiments.

This protocol was used to evaluate the time course of drug effects at each dose. For each drug dose, the protocols were repeated on three-to-four days in different rats, and the experimental points are given as the mean ± S.E.M. Statistical analysis was performed by ANOVA followed by ad-hoc Bonferroni's *t*-test to compare differences between drug doses at each time point. Differences were considered significant with P < 0.05. The dose–response relationships were drawn by reporting the mean TRR measured at each drug dose normalized with respect to the mean TRR measured with drug vehicle as a function of the drug dose. The relationships were fit with equation reported in figure legend.

### Sodium current measurement in HEK293 cells

Whole-cell sodium currents (I_Na_) were recorded with patch-clamp technique in HEK293 cells permanently transfected with the human skeletal muscle isoform of voltage-gated sodium channel, hNav1.4 (Desaphy et al., 2012). Sodium current recordings were performed at room temperature (20–22 °C) using an Axopatch 1D amplifier (Axon Instruments, Union City, CA, USA). Voltage clamp protocols and data acquisition were performed with pCLAMP 9.2 software (Axon Instruments) through a 12-bit A-D/D-A interface (Digidata 1340, Axon Instruments). Pipettes made with Corning 7052 glass (Garner Glass, Claremont, CA, USA) had resistance that ranged from 1 to 3 MΩ. Currents were low-pass filtered at 2 kHz (− 3 dB) by the four-pole Bessel filter of the amplifier and digitized at 10–20 kHz. After the rupture of patch membrane, a test pulse of − 30 mV from a holding potential of − 120 mV was applied to the cell until stabilization of sodium current amplitude and kinetics (typically 5 min). Only those data obtained from cells exhibiting voltage errors < 5 mV after series resistance compensation were considered for analysis. Little (< 5%) or no rundown was observed within the experiments.

### Drugs and solutions

Patch clamp pipette solution contained in mM: 120 CsF, 10 CsCl, 10 NaCl, 5 EGTA and 5 HEPES, and pH was set to 7.2 with CsOH. Bath solution for patch clamp recordings contained (in mM): 150 NaCl, 4 KCl, 2 CaCl2, 1 MgCl2, 5 HEPES and 5 glucose. The pH was set to 7.4 with NaOH. All the compounds were purchased from Sigma-Aldrich (Milan, Italy).

Mexiletine hydrochloride, anthracen-9-carboxylic acid (9AC), carbamazepine, carbamazepine 10,11-epoxide (epo-CBZ), propafenone hydrochloride, flecainide acetate, orphenadrine hydrochloride, were purchased from Sigma-Aldrich (Milan, Italy). Lubeluzole (R,S racemate) and riluzole were synthesized in the medicinal chemistry laboratories of our department as previously described (Bruno et al., 2006; Catalano et al., 2013; Desaphy et al., 2013c).

For patch-clamp experiments, the drugs were dissolved at the desired final concentration in external patch solution containing up to 1% dimethylsulfoxide. In our experimental conditions, we found no effect of 1% DMSO on sodium channels. Notwithstanding, control recordings were made in cells bathed with drug-free solutions containing the same amount of DMSO. The patched cell was continuously exposed to a stream of control (supplemented with DMSO as needed) or drug-supplemented bath solution flowing out from a plastic capillary. For in vivo experiments, a solution containing 2.4 g/l of 9AC was prepared each day in distilled water and 0.3% bicarbonate; the volume of i.p. injection was adjusted to get 30 mg/kg body weight (Desaphy et al., 2013b). Exploratory drugs were diluted at the desired concentration directly in physiological 0.9% NaCl saline for oral administration of a volume close to 1 ml.

## Results

### Antimyotonic effects of marketed drugs in the rat model of myotonia

Myotonia was induced in rats by intraperitoneal injection of 30 mg/kg 9AC and was evaluated by measuring the TRR. As detailed elsewhere, such a method allows a quantitative appreciation of reproducible myotonia (Desaphy et al., 2013b). Ten minutes after 9AC injection the TRR was dramatically prolonged from < 0.5 to ~ 2 s, and increased further to ~ 4 s 30 min after. Then the TRR decreased gradually over time, being ~ 1 s 3 h after 9AC. Drugs or vehicle was administrated orally to the rats 20 min after 9-AC injection. The TRR measured at each time point was normalized as a function of TRR measured 10 min after 9AC injection to compare myotonia between rats receiving the various drugs or vehicle.

**Fig. 1:**
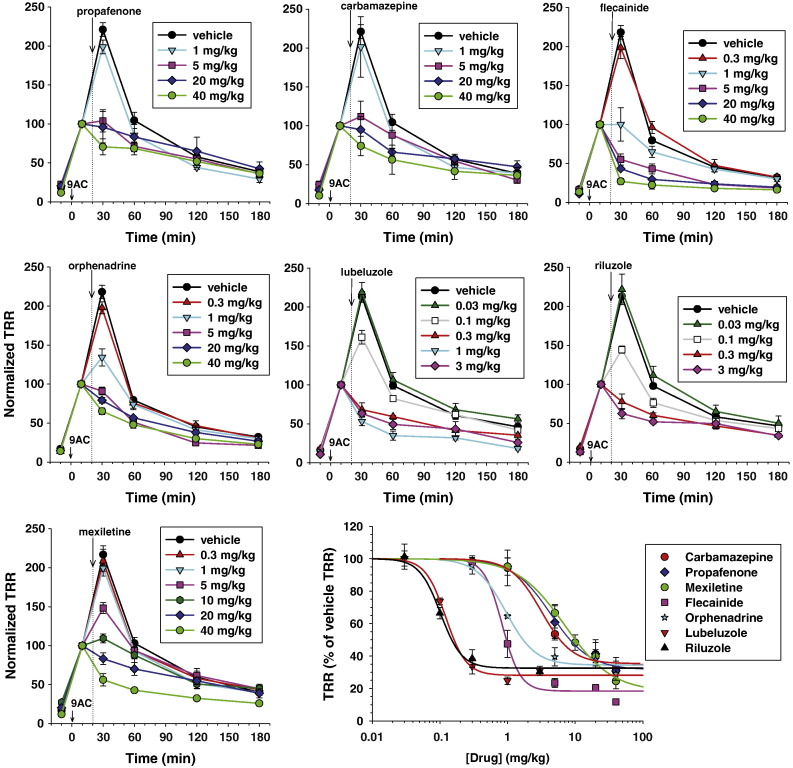
In vivo antimyotonic effects of exploratory drugs in the rat model of myotonia. The time course of normalized TRR values was determined in 9AC-treated rats receiving various doses of propafenone, carbamazepine, flecainide, orphenadrine, lubeluzole, riluzole, and mexiletine. Each data point is the mean ± S.E.M. calculated from 3-to-4 rats. Statistical analysis of differences between drug doses was performed at each time point with ANOVA followed by ad-hoc Bonferroni's *t*-test and results are reported in Supplementary Fig. S1. The dose–response curve was constructed for all the drugs by reporting the value of normalized TRR (expressed as percentage of the TRR calculated in rat receiving drug vehicle alone), measured 10 min after drug administration (i.e. 30 min after 9AC injection). The dose–response curve was fitted with equation TRR = min + (100 min) / [1 + exp([Drug] / ED_50_)^nH^], where [Drug] is the drug dose, ED_50_ value is the half-maximum efficient dose, nH is the slope factor, and min the curve baseline. Values of fit parameters ± the S.E. of the fit are given in Table 1.

The dose-dependent effects of the exploratory drugs are shown in Fig. 1 and statistical analysis is reported in Supplementary Fig. S1. Compare to vehicle, doses greater than 1 mg/kg of mexiletine, propafenone, or carbamazepine significantly reduced the TRR measured 30 min after 9AC. Both flecainide and orphenadrine exerted significant antimyotonic effect at doses superior to 0.3 mg/kg. Lubeluzole and riluzole resulted as the most potent drugs, with a significant reduction of TRR observed at the dose of 0.1 mg/kg. Drug effects were dose-dependent but saturated at the highest doses, without allowing a complete recovery of TRR. At the highest doses, the drugs still exerted a significant reduction of the TRR 60 min after 9AC injection (i.e. 40 min after drug administration), but little or no effect was observed then after. The TRR measured 30 min after 9AC injection in the presence of drugs was normalized with respect to that measured in the presence of vehicle to draw the dose–response curves for each drugs. The half-maximum efficient doses (ED_50_) are reported in Table 1. The drugs are distributed in three groups with ED_50_ values around 5 mg/kg (mexiletine, propafenone, carbamazepine), 1 mg/kg (orphenadrine and flecainide), and 0.1 mg/kg (lubeluzole and riluzole). The maximal effect reached about 80% of TRR reduction for mexiletine and flecainide, and ~ 70% for all the other drugs.

The dose-dependent effects of the exploratory drugs are shown in Fig. 1 and statistical analysis is reported in Supplementary Fig. S1. Compare to vehicle, doses greater than 1 mg/kg of mexiletine, propafenone, or carbamazepine significantly reduced the TRR measured 30 min after 9AC. Both flecainide and orphenadrine exerted significant antimyotonic effect at doses superior to 0.3 mg/kg. Lubeluzole and riluzole resulted as the most potent drugs, with a significant reduction of TRR observed at the dose of 0.1 mg/kg. Drug effects were dose-dependent but saturated at the highest doses, without allowing a complete recovery of TRR. At the highest doses, the drugs still exerted a significant reduction of the TRR 60 min after 9AC injection (i.e. 40 min after drug administration), but little or no effect was observed then after. The TRR measured 30 min after 9AC injection in the presence of drugs was normalized with respect to that measured in the presence of vehicle to draw the dose–response curves for each drugs. The half-maximum efficient doses (ED_50_) are reported in Table 1. The drugs are distributed in three groups with ED_50_ values around 5 mg/kg (mexiletine, propafenone, carbamazepine), 1 mg/kg (orphenadrine and flecainide), and 0.1 mg/kg (lubeluzole and riluzole). The maximal effect reached about 80% of TRR reduction for mexiletine and flecainide, and ~ 70% for all the other drugs.

### Block of skeletal muscle Nav1.4 channels by antimyotonic drugs

**Fig. 2:**
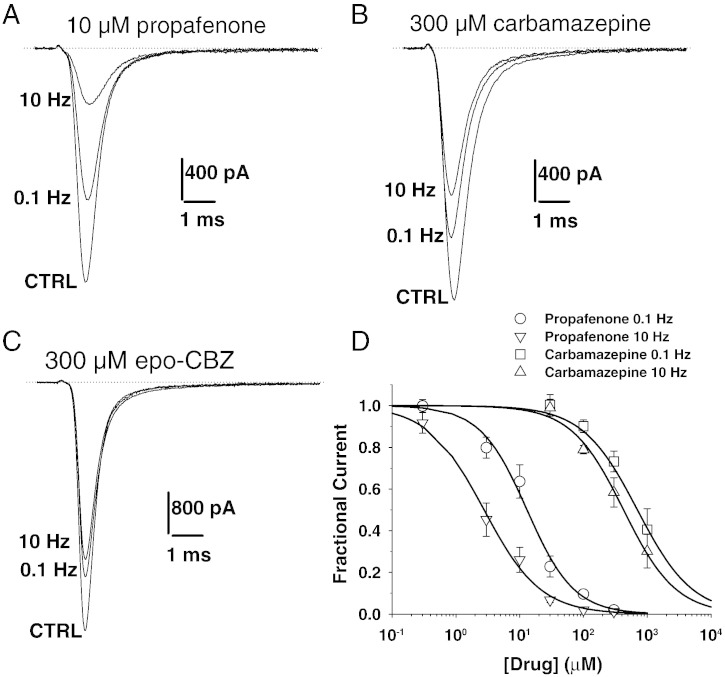
Effects of propafenone, carbamazepine, and carbamazepine-10,11-epoxide, on hNav1.4 sodium channel subtype. (A–C) Sodium currents were measured in HEK293 cells permanently transfected with the human skeletal muscle hNav1.4 isoform using the whole-cell patch-clamp method. Currents were elicited by depolarizing the cells for 20 ms at − 30 mV from a holding potential of − 120 mV, every 10 or 0.1 s (i.e. 0.1 Hz or 10 Hz stimulation frequency, respectively). Sodium current traces recorded in representative cells are shown in control condition (CTRL) and after acute application of 10 μM propafenone, 300 μM carbamazepine, or 300 μM carbamazepine-10,11-epoxide, at 0.1 and 10 Hz. (D) Concentration–response relationships were constructed at 0.1 and 10 Hz frequency stimulation for propafenone and carbamazepine. Each point is the mean ± S.E.M. from at least 3 cells. The relationships were fitted with equation [I_DRUG_ / I_CTRL_ = 1 / {1 + ([DRUG] / IC_50_)^nH^}], where IC_50_ value is the half-maximum inhibitory concentration and nH is the slope factor. The nH values were comprised in between 0.8 and 1.2. The calculated IC_50_ ± S.E. of the fit is reported in Table 2.

For many of the tested drugs, the block of heterologously expressed hNav1.4 channels has been evaluated by patch-clamp technique in previous studies, using rigorously identical voltage clamp protocols. Sodium currents were elicited by depolarizing the cells from the holding potential of − 120 mV to a 30 ms-long test pulse at − 30 mV. Sodium current inhibition by drugs was measured at both 0.1 and 10 Hz stimulation frequencies (Desaphy et al., 2004, 2009, 2012, 2013c). Here we completed these experiments for propafenone and carbamazepine (CBZ). Examples of sodium current traces are shown in Fig. 2, which were recorded before (CTRL) and at the steady-state of drug block, i.e. 3 min after drug application at 0.1 Hz, and between the 100th and 110th pulse at 10 Hz. Propafenone, at the concentration of 10 μM, reduced peak current by 36 ± 8% at 0.1 Hz and by 74 ± 6% at 10 Hz (n = 4) (Fig. 2A). Carbamazepine, at the concentration of 300 μM reduced peak current by 27 ± 5% at 0.1 Hz and by 42 ± 7% at 10 Hz (n = 4) (Fig. 2B). The concentration–response relationships were fitted to evaluate the half-maximum inhibitory concentration (Fig. 2C). The IC_50_ values for CBZ and propafenone are reported in Table 2, together with the IC_50_ values previously determined for the other drugs in exam. At 0.1 Hz stimulation frequency, the less potent in blocking sodium currents was carbamazepine, followed by mexiletine, orphenadrine and flecainide, riluzole and lubeluzole, while the more potent was propafenone. The drugs presented with different use-dependent properties, thus the order of potency at 10 Hz was, from the less to the more potent, CBZ < riluzole ~ flecainide < mexiletine < orphenadrine < propafenone < lubeluzole.

It has been proposed that the CBZ metabolite, carbamazepine-10,11-epoxide (epo-CBZ), may be able to exert significant pharmacological effect through sodium channel blockade (McLean and Macdonald, 1986; Yoshimura et al., 1998). In an attempt to find an explanation for the disconnection between CBZ effects in vitro and in vivo, we tested epo-CBZ on hNav1.4 sodium currents (Fig. 2D). Sodium current inhibition exerted by 300 μM of the metabolite at 0.1 Hz (22.1 ± 9%, n = 4) and 10 Hz (33.8 ± 13%, n = 4) was not significantly different from that exerted by the same concentration of CBZ (P > 0.05, unpaired Student's *t* test). The dose–response curve was not constructed for epo-CBZ, since the metabolite was poorly soluble at concentrations greater than 300 μM.

**Fig. 3:**
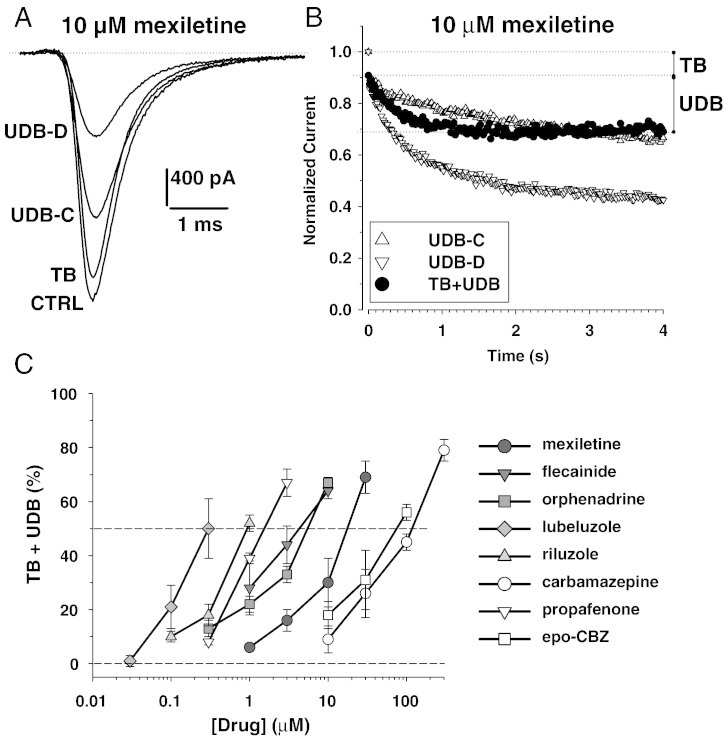
Effects of exploratory drugs on hNav1.4 sodium channel subtype in myotonia-like conditions. Sodium currents were elicited in HEK293 cells permanently transfected with the human skeletal muscle hNav1.4 isoform using the whole-cell patch-clamp method. To mimic a myotonia condition, the cells were depolarized for 5 ms (action potential duration) at − 30 mV from a holding potential of − 90 mV (sarcolemma resting membrane potential), the stimulation frequency of 50 Hz (myotonic run frequency). (A) Sodium current traces recorded in a representative cell are shown in control condition at 0.1 Hz (CTRL) and 50 Hz (use-dependent block in control, UDB-C) and after acute application of 10 μM mexiletine at 0.1 Hz (tonic block exerted by the drug, TB) and 50 Hz (use-dependent block observed in the presence of drug, UDB-D). (B) Time course of sodium current peak amplitude reduction induced by 50 Hz stimulation in the absence of drug (UDB-C, triangles) and in the presence of 10 μM mexiletine (UDB-D, inverted triangles). The net reduction induced by the drug was obtained by subtraction of UDB-C to UDB-D and addition of TB. The relationships represent the means from 3 cells; S.E.M. is not reported for improving clarity of the graph. (C) Concentration–response relationships were constructed for all the exploratory drugs following the protocol shown in (B). Each point is the mean ± S.E.M. from at least 3 cells. The half-maximum inhibitory concentration (IC_50_) values were approximated as the intercept of the relationships with 50% of (TB + UDB), and are reported in Table 2.

We further evaluated the effects of drugs on sodium currents using a voltage-clamp protocol that mimics more closely the myotonic condition (Fig. 3). The holding potential was fixed to − 90 mV (near the resting membrane potential of muscle fibers) and the sodium currents were elicited by test pulses at − 30 mV applied at the stimulation frequency of 50 Hz (myotonic discharges have frequencies ranging from tens to hundreds of Hz). The test pulses were 5 ms long, recalling the action potential duration. In these conditions, a use-dependent reduction of sodium currents developed in the absence of drugs (UDB-C, 33 ± 5% reduction, n = 10). A representative example of 10 μM-mexiletine effects is shown in Fig. 3A. In this cell, the UDB-C was 34.6%. At the frequency of 0.1 Hz, a little tonic block was observed after application of mexiletine (TB, 9.5% reduction). In the presence of the drug, a huge use-dependent reduction was measured at 50 Hz (UDB-D, 66.7% reduction). The time course of use-dependent effects in the presence and absence of 10 μM mexiletine (n = 3), as well as the tonic and use-dependent block (TB + UDB) directly attributable to the drug, which was obtained by subtraction is illustrated in Fig. 3B. These latter values were calculated for the entire set of drugs and reported as a function of drug concentration (Fig. 3C). Interestingly, both carbamazepine and riluzole that showed little use-dependence between 0.1 and 10 Hz stimulation at the holding potential of − 120 mV, exerted a significant use-dependent block in myotonia-like conditions. The IC_50_ values, approximated as the intercept of the relationships with 50% TB + UDB, are reported in Table 2. In these conditions, the order of potency was, from the less to the more potent, CBZ < epo-CBZ < mexiletine < orphenadrine ~ flecainide < propafenone < riluzole < lubeluzole.

## Discussion

This study demonstrates that many sodium channel blockers can exert antimyotonic activity in vivo. The rat model of myotonia was previously validated by testing the antimyotonic activity of mexiletine and β-adrenergic drugs able to block sodium channels or not (Desaphy et al., 2003, 2013b). In this previous study, propranolol and clenbuterol were shown to exert significant hNav1.4 sodium channel blockade in vitro and antimyotonic activity in the rat. Nevertheless, the ED_50_ values for both adrenergic drugs were close to 20 mg/kg in the rat, which is greater than the usual clinical dose in humans. All the sodium channel blockers tested in the present study were more potent than mexiletine in vivo. In an attempt to translate the effects measured in the rat model to the clinics, we compared the ED_50_ values measured in rats to the reported antimyotonic dose in humans (Table 1). During the clinical trial in NDM, mexiletine was given to patients at the daily dose of 600 mg (Statland et al., 2012), which corresponds to 8.6 mg/kg for 70 kg body weight and is close to the ED_50_ value for antimyotonic activity in the rat (7 mg/kg). A similar relationship applies to propafenone (Alfonsi et al., 2007), while the ED_50_ value calculated in the rat was three fold minor than the daily dose reported in humans for carbamazepine (Sechi et al., 1983) and flecainide (Desaphy et al., 2013a). There is no report about the use of orphenadrine and riluzole in myotonia therapy, but these drugs are used in humans for other indications. Interestingly, the ED_50_ value for antimyotonic activity in the rat model was six-to-ten fold lower than the usual dose in humans, suggesting that these drugs can likely be used safely by myotonic patients (Table 1). Lubeluzole has been used in humans as a neuroprotectant in stroke, but clinical trials failed to confirm its usefulness in this setting (Gandolfo et al., 2002). At the clinical dose of 10 mg/day (close to the antimyotonic ED_50_ value in rats), lubeluzole was however associated with a significant prolongation of QT interval (> 450 ms); thus lubeluzole safety profile deserves further in-depth studies before to use it in myotonic patients.

**Fig. 4:**
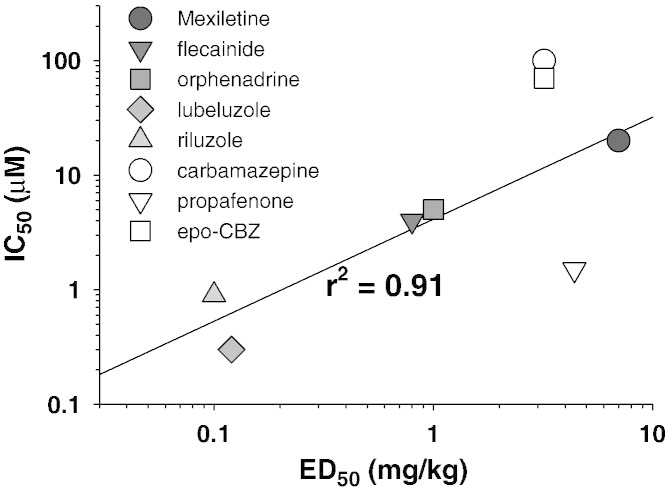
Relationship between in vitro hNav1.4 sodium channel inhibition and in vivo antimyotonic activity in the rat model. The IC_50_ values for sodium current inhibition calculated in vitro in myotonia-like conditions (calculated as in Fig. 3) are reported versus the ED_50_ value for in vivo antimyotonic activity in the rat model (measured as in Fig. 1). For carbamazepine-10,11-epoxide (epo-CBZ), the ED_50_ value was measured for carbamazepine. The linear regression considering all the drugs (but epo-CBZ) had a coefficient r^2^ = 0.52 (not shown). The linear regression of all drugs except epo-CBZ, carbamazepine, and propafenone, shows an r^2^ coefficient of 0.91.

In the present study, we were also interested at evaluating the relationship between sodium channel inhibition in vitro and antimyotonic activity in vivo. Poor regression coefficients were obtained when plotting the ED_50_ values obtained in vivo in the rat model versus the IC50 values measured at 0.1 or 10 Hz (not shown). In contrast, a good linear correlation (r^2^ = 0.91) was obtained between the IC_50_ values determined in myotonia-like conditions in vitro and the ED_50_ values (Fig. 4), when considering all drugs but propafenone and carbamazepine (or its metabolite). Indeed propafenone showed a weak antimyotonic activity in vivo with respect to its activity on sodium channels in vitro, whereas carbamazepine appeared more efficient in vivo than expected from in vitro experiments. The apparent discrepancies for these two drugs are probably related to in vivo pharmacokinetics or pharmacodynamics. In this regard, although the metabolite epo-CBZ exerted a slightly greater sodium channel blockade than carbamazepine, this appears insufficient to account for the disconnection between in vivo and in vitro effects. On the other hand, although the maximal dose recommended in adults is 600 mg for both mexiletine and carbamazepine, the expected therapeutic range of plasma concentration is in between 4 and 12 μg/ml for carbamazepine and 0.5–2 μg/ml for mexiletine, suggesting that bioavailability of carbamazepine is about 6 fold superior. A carbamazepine concentration of 12 μg/ml corresponds to 51 μM, which is close to the IC_50_ of carbamazepine (100 μM) and its active metabolite (70 μM) calculated in vitro. This observation suggests that pharmacokinetics may contribute to the poor correlation between the measured in vivo and in vitro effects of carbamazepine. Regarding propafenone, besides sodium channel block, the drug is known to inhibit β-adrenergic receptors (Burnett et al., 1988). The β-adrenergic agonists are known to stimulate the Na^+^, K^+^ pump, thereby inducing hyperpolarization and increasing uptake of K^+^ in skeletal muscle in vivo (Flatman and Clausen, 1976; Ford et al., 1995). It is widely acknowledged that chloride channel myotonia develops because the low chloride conductance in myotonia congenita patients cannot counterbalance the depolarization stemming from K^+^ accumulation in T-tubules (Adrian and Bryant, 1974; Cannon, 2001; Clausen, 2013). We may thus hypothesize that inhibition of β-adrenergic signaling by propafenone may favor K^+^ accumulation in T-tubules, thereby balancing the beneficial effects of sodium channel inhibition on myotonia. It is worth to note however that a recent study performed on isolated muscles of myotonic mice suggested that depolarization induced by increasing extracellular K^+^ concentration may contrast myotonia (Hoppe et al., 2013). Further studies are warranted to clarify the apparent discrepancies.

We can note that many sodium channel blockers display little selectivity among neuronal and skeletal muscle channels. Although we cannot exclude inhibition of neuronal sodium channels by the tested drugs in our in vivo model, it is unlikely that such a block is determinant for the antimyotonic activity, because myotonic hyperexcitability is intrinsic to muscle fibers.

In conclusion, the antimyotonic activity of drugs in vivo is well correlated with their potency in blocking sodium channels in vitro when using a myotonia-like condition. Significant differences may be present, such as for propafenone and carbamazepine, likely due to pharmacokinetics and multiple targets. By comparing the antimyotonic dose calculated in rats and the current clinical dose in humans, it appears that all the tested drugs may be used safely for the treatment of human myotonia (with some concern regarding lubeluzole). The results confirm the antimyotonic activity of carbamazepine; this drug is already considered by many as a second choice after mexiletine in adults. Carbamazepine is sometimes preferred for treatment of younger patients, because drug dosage and safety profile are better defined in children. For instance, the drug has shown great efficacy in severe neonatal episodic laryngospasm, a life-threatening form of sodium channel myotonia (Caietta et al., 2013; Lion-Francois et al., 2010). Flecainide and propafenone are also already used by myotonic patients carrying specific sodium channel mutations, who did not get satisfactory response from mexiletine (Alfonsi et al., 2007; Desaphy et al., 2013a; Rosenfeld et al., 1997). The present study suggests that these drugs may be efficient also in patients carrying chloride channel mutations. In addition the results indicate that orphenadrine and riluzole merit attention to increase the number of therapeutic options. We previously demonstrated that the sodium channel mutations may significantly decrease channel sensitivity to mexiletine, while effects of flecainide are preserved, thereby opening the way toward a pharmacogenetics strategy (Desaphy et al., 2001, 2004, 2013a). It would be interesting to verify whether hNav1.4 mutations modify channel sensitivity to the other examined drugs. Considering the occurrence of mexiletine non-responders and the limited tolerability of mexiletine in other patients, this study sets the basis for the definition of an arsenal of alternative drugs, which may prove useful to increase the quality of life of individuals suffering from non-dystrophic myotonia. While the rat model of myotonia congenita offers a valuable preclinical platform for testing antimyotonic drug candidates, further clinical trials are warranted to confirm these results (Trivedi et al., 2013a).

The following are the supplementary data related to this article.

Fig. S1 Statistical analysis of results reported in Fig. 1 was performed for each drug at each time point by ANOVA followed by ad-hoc Bonferroni's *t*-test. In the following tables only significant statistical results obtained by ANOVA (at least P < 0.05) are reported. k is the number of tested drug doses, N is the total number of tested rats. In the columns the direct comparison between two doses using Bonferroni's *t*-test (at least P < 0.05) is reported. NS means not significant (P > 0.05).

Supplementary data to this article can be found online at http://dx.doi.org/10.1016/j.expneurol.2014.02.023.

## Conflict of interest statement

The authors declare no conflict of interest.

## Authorship

Conception and design of the study: JFD and DCC.

Acquisition, analysis and interpretation of the data: JFD, RC, and TC.

Writing the manuscript: JFD.

Critically revising the manuscript: DCC.

## Figures and Tables

**Table 1:** In vivo antimyotonic potency in the rat model and usual clinical doses in humans.

Drug	ED_50_ (mg/kg)	nH	Min	Usual clinical dose in humans
Mexiletine	7.0 ± 1.4	1.2 ± 0.2	18.0 ± 7.3	600 mg/day → 8.6 mg/kg/day
Propafenone	4.4 ± 1.1	1.5 ± 0.6	31.4 ± 6.9	325 mg/day → 4.6 mg/kg/day
Carbamazepine	3.2 ± 0.7	1.9 ± 0.7	35.3 ± 4.5	600 mg/day → 8.6 mg/kg/day
Flecainide	0.8 ± 0.1	3.2 ± 1.8	18.4 ± 3.5	200 mg/day → 3 mg/kg/day
Orphenadrine	1.0 ± 0.2	2.0 ± 0.8	34.4 ± 3.3	400 mg/day → 5.7 mg/kg/day (myorelaxant)
Lubeluzole	0.12 ± 0.01	2.8 ± 0.8	28.1 ± 2.4	10 mg/day → 0.14 mg/kg/day (neuroprotectant)
Riluzole	0.10 ± 0.01	2.8 ± 1.4	32.5 ± 4.5	100 mg/day → 1.4 mg/kg/day (Amyotrophic Lateral Sclerosis)

The half-maximum efficient dose (ED_50_), the slope factor (nH), and the minimal value of TRR (min) were calculated from the fit of dose–response relationships obtained in the rat model of myotonia as shown in Fig. 1. Each value is reported ± S.E. of the fit. The reported clinical dose used in myotonic patients (references are given in the text) or other clinical indications (in brackets) are expressed in mg/kg/day, considering a body weight of 70 kg.

**Table 2:** In vitro potency in sodium channel block.

Drug	IC_50_ (μM)		
	0.1 Hz, hp − 120 mV	10 Hz, hp − 120 mV	50 Hz, hp − 90 mV
Mexiletine	246 ± 15	24 ± 2	20
Propafenone	13 ± 1	2.8 ± 0.3	1.5
Carbamazepine	701 ± 183	425 ± 64	100
epo-CBZ	n.d.	n.d.	70
Orphenadrine	93 ± 12	13 ± 1	5
Flecainide	84 ± 17	37 ± 6	4
Lubeluzole	31 ± 5	1.7 ± 0.2	0.3
Riluzole	50 ± 8	43 ± 3	0.9

The half-maximum inhibitory concentration (IC_50_) values at the holding potential (hp) of − 120 mV were calculated from the fit of concentration–response relationships shown in Fig. 2. Each value is reported ± S.E. of the fit. The IC_50_ values for epo-CBZ were not determined. At the hp of − 90 mV, the IC_50_ values were approximated by eyes as the intercept of the concentration–response relationships with 50% of TB + UDB, as shown in Fig. 3C.

## References

[bb0005] Adrian R.H., Bryant S.H. (1974). On the repetitive discharge in myotonic muscle. J. Physiol..

[bb0010] Alfonsi E., Merlo I.M., Tonini M., Ravaglia S., Brugnoni R., Gozzini A., Moglia A. (2007). Efficacy of propafenone in paramyotonia congenita. Neurology.

[bb0015] Bruno C., Carocci A., Catalano A., Cavalluzzi M.M., Corbo F., Franchini C., Lentini G., Tortorella V. (2006). Facile, alternative route to lubeluzole, its enantiomer, and the racemate. Chirality.

[bb0020] Burnett D.M., Gal J., Zahniser N.R., Nies A.S. (1988). Propafenone interacts stereoselectively with beta 1- and beta 2-adrenergic receptors. J. Cardiovasc. Pharmacol..

[bb0025] Caietta E., Milh M., Sternberg D., Lépine A., Boulay C., McGonigal A., Chabrol B. (2013). Diagnosis and outcome of SCN4A-related severe neonatal episodic laryngospasm (SNEL): 2 new cases. Pediatrics.

[bb0030] Cannon S.C. (2001). Voltage-gated ion channelopathies of the nervous system. Clin. Neurosci. Res..

[bb0035] Catalano A., Carocci A., Defrenza I., Muraglia M., Carrieri A., Van Bambeke F., Rosato A., Corbi F., Franchini C. (2013). 2-Aminobenzothiazole derivatives: search for new antifungal agents. Eur. J. Med. Chem..

[bb0040] Clausen T.B. (2013). Excitation-induced exchange of Na^+^, K^+^, and Cl^−^ in rat EDL muscle in vitro and in vivo: physiology and pathophysiology. J. Gen. Physiol..

[bb0045] Conte Camerino D., Tricarico D., Desaphy J.-F. (2007). Ion channel pharmacology. Neurotherapeutics.

[bb0050] Desaphy J.-F., Conte Camerino D., Tortorella V., De Luca A. (1999). Effect of mexiletine on sea anemone toxin-induced non-inactivating sodium channels of rat skeletal muscle: a model of sodium channel myotonia. Neuromuscul. Disord..

[bb0070] Desaphy J.-F., De Luca A., Tortorella P., De Vito D., George A.L., Conte Camerino D. (2001). Gating of myotonic Na channel mutants defines the response to mexiletine and a potent derivative. Neurology.

[bb0090] Desaphy J.-F., Pierno S., De Luca A., Didonna M.P., Conte Camerino D. (2003). Different ability of clenbuterol and salbutamol to block sodium channels predicts their therapeutic use in muscle excitability disorders. Mol. Pharmacol..

[bb0065] Desaphy J.-F., De Luca A., Didonna M.P., George A.L., Conte Camerino D. (2004). Different flecainide sensitivity of hNav1.4 channels and myotonic mutants explained by state-dependent block. J. Physiol..

[bb0080] Desaphy J.-F., Dipalma A., De Bellis M., Costanza T., Gaudioso C., Delmas P., George A.L., Conte Camerino D. (2009). Involvement of voltage-gated sodium channels blockade in the analgesic action of orphenadrine. Pain.

[bb0075] Desaphy J.-F., Dipalma A., Costanza T., Carbonara R., Dinardo M.M., Catalano A., Carocci A., Lentini G., Franchini C., Conte camerino D. (2012). Molecular insights into the local anesthetic receptor within voltage-gated sodium channels using hydroxylated analogs of mexiletine. Front. Pharmacol..

[bb0085] Desaphy J.-F., Modoni A., Lomonaco M., Conte Camerino D. (2013). Dramatic improvement of myotonia permanens with flecainide: a two-case report of a possible bench-to-bedside pharmacogenetics strategy. Eur. J. Clin. Pharmacol..

[bb0060] Desaphy J.-F., Costanza T., Carbonara R., Conte Camerino D. (2013). In vivo evaluation of antimyotonic efficacy of β-adrenergic drugs in a rat model of myotonia. Neuropharmacology.

[bb0055] Desaphy J.-F., Carbonara R., Costanza T., Lentini G., Cavalluzzi M.M., Bruno C., Franchini C., Conte Camerino D. (2013). Molecular dissection of lubeluzole use-dependent block of voltage-gated sodium channels discloses new therapeutic potentials. Mol. Pharmacol..

[bb0095] Flatman J.A., Clausen T. (1976). Combined effects of adrenaline and insulin on active electrogenic Na^+^–K^+^ transport in rat soleus muscle. Nature.

[bb0100] Ford G.A., Dachman W.D., Blaschke T.F., Hoffman B.B. (1995). Effect of aging on beta 2-adrenergic receptor-stimulated flux of K^+^, PO_4_, FFA, and glycerol in human forearms. J. Appl. Physiol..

[bb0105] Gandolfo C., Sandercock P., Conti M. (2002). Lubeluzole for acute ischaemic stroke. Cochrane Database Syst. Rev..

[bb0110] Hoppe K., Lehmann-Horn F., Chaiklieng S., Jurkat-Rott K., Adolph O., Klingler W. (2013). In vitro muscle contracture investigations on the malignant hyperthermia like episodes in myotonia congenita. Acta Anaesthesiol. Scand..

[bb0115] Lion-Francois L., Mignot C., Vicart S., Manel V., Sternberg D., Landrieu P., Lesca G., Broussolle E., Billette de Villemeur T., Napuri S., des Portes V., Fontaine B. (2010). Severe neonatal episodic laryngospasm due to de novo SCN4A mutations: a new treatable disorder. Neurology.

[bb0120] Logigian E.L., Martens W.B., Moxley R.T., McDermott M.P., Dilek N., Wiegner A.W., Pearson A.T., Barbieri C.A., Annis C.L., Thornton C.A., Moxley R.T. (2010). Mexiletine is an effective antimyotonia treatment in myotonic dystrophy type 1. Neurology.

[bb0125] Lyons M.J., Duron R., Molinero I., Sangiuolo F., Holden K.R. (2010). Novel CLCN1 mutation in carbamazepine-responsive myotonia congenita. Pediatr. Neurol..

[bb0130] Matthews E., Fialho D., Tan S.V., Venance S.L., Cannon S.C., Sternberg D., Fontaine B., Amato A.A., Barohn R.J., Griggs R.C., Hanna M.G., CINCH investigators (2010). The non-dystrophic myotonias: molecular pathogenesis, diagnosis and treatment. Brain.

[bb0135] McLean M.J., Macdonald R.L. (1986). Carbamazepine and 10,11-epoxycarbamazepine produce use- and voltage-dependent limitation of rapidly firing action potentials of mouse central neurons in cell culture. J. Pharmacol. Exp. Ther..

[bb0140] Rayan D.R., Hanna M.G. (2010). Skeletal muscle channelopathies: nondystrophic myotonias and periodic paralysis. Curr. Opin. Neurol..

[bb0145] Rosenfeld J., Sloan-Brown K., George A.L. (1997). A novel muscle sodium channel mutation causes painful congenital myotonia. Ann. Neurol..

[bb0150] Savitha M.R., Krishnamurthy B., Hyderi A., Farhan-Ul-Haque, Ramachandra N.B. (2006). Myotonia congenita — a successful response to carbamazepine. Indian J. Pediatr..

[bb0155] Sechi G.P., Traccis S., Durelli L., Monaco F., Mutani R. (1983). Carbamazepine versus diphenylhydantoin in the treatment of myotonia. Eur. Neurol..

[bb0160] Statland J.M., Bundy B.N., Wang Y., Rayan D.R., Trivedi J.R., Sansone V.A., Salajeqheh M.K., Venance S.L., Ciafaloni E., Matthews E., Meola G., Herbelin L., Griggs R.C., Barohn R.J., Hanna M.G., Consortium for Clinical Investigation of Neurologic Channelopathies (2012). Mexiletine for symptoms and signs of myotonia in nondystrophic myotonia: a randomized controlled trial. JAMA.

[bb0165] Trivedi J.R., Bundy B., Statland J., Salajeqheh M., Rayan D.R., Venance S.L., Wang Y., Fialho D., Matthews E., Cleland J., Gorham N., Herbelin L., Cannon S., Amato A., Griggs R.C., Hanna M.G., Barohn R.J., CINCH consortium (2013). Non-dystrophic myotonia: prospective study of objective and patient reported outcomes. Brain.

[bb0170] Trivedi J.R., Cannon S.C., Griggs R.C. (2013). Nondystrophic myotonia: challenges and future directions. Exp. Neurol..

[bb0175] Wang G.K., Russell C., Wang S.Y. (2004). Mexiletine block of wild-type and inactivation-deficient human skeletal muscle hNav1.4 Na^+^ channels. J. Physiol..

[bb0180] Yoshimura R., Yanagihara N., Terao T., Minami K., Toyohira Y., Ueno S., Uezono Y., Abe K., Izumi F. (1998). An active metabolite of carbamazepine, carbamazepine-10,11-epoxide, inhibits ion channel-mediated catecholamine secretion in cultures bovine adrenal medullary cells. Psychopharmacology.

