# Supplementary material for: Preclinical evaluation of marketed sodium channel blockers in a rat model of myotonia discloses promising antimyotonic drugs
Source: Exp Neurol. 2014 May;255(100):96–102. doi: 10.1016/j.expneurol.2014.02.023 (PMC4004800; doi:10.1016/j.expneurol.2014.02.023)
Supplement: Fig. S1 — Statistical analysis of results reported in Fig. 1 was performed for each drug at each time point by ANOVA followed by ad-hoc Bonferroni's t-test. In the following tables only significant statistical results obtained by ANOVA (at least P < 0.05) are reported. k is the number of tested drug doses, N is the total number of tested rats. In the columns the direct comparison between two doses using Bonferroni's t-test (at least P < 0.05) is reported. NS means not significant (P > 0.05). [file mmc1.pdf]

Preclinical evaluation of marketed sodium channel blockers in a rat model of myotonia discloses promising antimyotonic drugs. Desaphy, Carbonara, Costanza, and Conte Camerino

FIGURE S1. Statistical analysis of results reported in Figure 1 was performed for each drug at each time point by ANOVA followed by ad-hoc Bonferroni's t-test. In the following tables are reported only significant statistical results obtained by ANOVA (at least P<0.05). k is the number of tested drug doses, N is the total number of tested rats. In the columns are reported the direct comparison between two doses using Bonferroni's t-test (at least P<0.05). NS means not significant (P>0.05).

**Mexiletine**, time point +30			**ANOVA**: k-1 = 6, N-k = 48, F=37.5367, P<0.0001			
**mg/kg**	**0 (vehicle)**	**0.3**	**1**	**5**	**10**	**20**
**0.3**	NS					
**1**	NS	NS				
**5**	P<0.0001	P<0.01	P<0.01			
**10**	P<0.0001	P<0.0001	P<0.0001	P<0.05		
**20**	P<0.0001	P<0.0001	P<0.0001	P<0.0001	NS	
**40**	P<0.0001	P<0.0001	P<0.0001	P<0.0001	P<0.05	NS

**Mexiletine**, time point +60			**ANOVA**: k-1 = 6, N-k = 48, F=2.4623, P<0.05			
**mg/kg**	**0 (vehicle)**	**0.3**	**1**	**5**	**10**	**20**
**0.3**	NS					
**1**	NS	NS				
**5**	NS	NS	NS			
**10**	NS	NS	NS	NS		
**20**	P<0.05	NS	NS	NS	NS	
**40**	P<0.01	P<0.05	P<0.05	P<0.05	P<0.05	NS

**Flecainide**, time point +30		**ANOVA**: k-1 = 5, N-k = 26, F=40.7437, P<0.0001			
**mg/kg**	**0 (vehicle)**	**0.3**	**1**	**5**	**20**
**0.3**	NS				
**1**	P<0.0001	P<0.001			
**5**	P<0.0001	P<0.0001	NS		
**20**	P<0.0001	P<0.0001	P<0.05	NS	
**40**	P<0.0001	P<0.0001	P<0.01	NS	NS

**Flecainide**, time point +60		**ANOVA**: k-1 = 5, N-k = 26, F=20.1821, P<0.0001			
**mg/kg**	**0 (vehicle)**	**0.3**	**1**	**5**	**20**
**0.3**	NS				
**1**	NS	P<0.01			
**5**	P<0.0001	P<0.0001	P<0.05		
**20**	P<0.0001	P<0.0001	P<0.01	NS	
**40**	P<0.0001	P<0.0001	P<0.001	NS	NS

**Flecainide**, time point +120		**ANOVA**: k-1 = 5, N-k = 26, F=8.599, P<0.0001			
**mg/kg**	**0 (vehicle)**	**0.3**	**1**	**5**	**20**
**0.3**	NS				
**1**	NS	NS			
**5**	P<0.001	P<0.01	P<0.01		
**20**	P<0.001	P<0.01	P<0.05	NS	
**40**	P<0.0001	P<0.001	P<0.01	NS	NS

**Flecainide**, time point +180		**ANOVA**: k-1 = 5, N-k = 26, F=5.7185, P<0.002			
**mg/kg**	**0 (vehicle)**	**0.3**	**1**	**5**	**20**
**0.3**	NS				
**1**	NS	NS			
**5**	P<0.01	P<0.05	P<0.05		
**20**	P<0.01	P<0.05	P<0.05	NS	
**40**	P<0.001	P<0.01	P<0.05	NS	NS

**CBZ**, time point +30		**ANOVA**: k-1 = 4, N-k = 19, F=17.3347, P<0.0001		
**mg/kg**	**0 (vehicle)**	**1**	**5**	**20**
**1**	NS			
**5**	P<0.0001	P<0.01		
**20**	P<0.0001	P<0.01	NS	
**40**	P<0.0001	P<0.001	NS	NS

**orphenadrine**, time point +30		**ANOVA**: k-1 = 5, N-k = 26, F=30.9647, P<0.0001			
**mg/kg**	**0 (vehicle)**	**0.3**	**1**	**5**	**20**
**0.3**	NS				
**1**	P<0.0001	P<0.01			
**5**	P<0.0001	P<0.0001	NS		
**20**	P<0.0001	P<0.0001	P<0.05	NS	
**40**	P<0.0001	P<0.0001	P<0.01	NS	NS

**orphenadrine**, time point +60		**ANOVA**: k-1 = 5, N-k = 26, F=6.1174, P<0.003			
**mg/kg**	**0 (vehicle)**	**0.3**	**1**	**5**	**20**
**0.3**	NS				
**1**	NS	NS			
**5**	P<0.01	P<0.05	P<0.05		
**20**	P<0.01	NS	NS	NS	
**40**	P<0.001	P<0.01	P<0.05	NS	NS

**orphenadrine**, time point +120		**ANOVA**: k-1 = 5, N-k = 26, F=3.7577, P<0.03			
**mg/kg**	**0 (vehicle)**	**0.3**	**1**	**5**	**20**
**0.3**	NS				
**1**	NS	NS			
**5**	P<0.01	P<0.01	P<0.01		
**20**	NS	NS	NS	NS	
**40**	P<0.01	P<0.01	NS	NS	NS

**lubeluzole**, time point +30		**ANOVA**: k-1 = 5, N-k = 24, F=53.9369, P<0.0001			
**mg/kg**	**0 (vehicle)**	**0.03**	**0.1**	**0.3**	**1**
**0.03**	NS				
**0.1**	P<0.01	P<0.01			
**0.3**	P<0.0001	P<0.0001	P<0.0001		
**1**	P<0.0001	P<0.0001	P<0.0001	NS	
**3**	P<0.0001	P<0.0001	P<0.0001	NS	NS

**lubeluzole**, time point +60		**ANOVA**: k-1 = 5, N-k = 24, F=16.0482, P<0.0001			
**mg/kg**	**0 (vehicle)**	**0.03**	**0.1**	**0.3**	**1**
**0.03**	NS				
**0.1**	NS	NS			
**0.3**	P<0.001	P<0.001	NS		
**1**	P<0.0001	P<0.0001	P<0.001	NS	
**3**	P<0.0001	P<0.0001	P<0.05	NS	NS

**lubeluzole**, time point +120		**ANOVA**: k-1 = 5, N-k = 24, F=3.8042, P<0.02			
**mg/kg**	**0 (vehicle)**	**0.03**	**0.1**	**0.3**	**1**
**0.03**	NS				
**0.1**	NS	NS			
**0.3**	P<0.01	P<0.01	NS		
**1**	P<0.01	P<0.01	P<0.05	NS	
**3**	NS	P<0.05	NS	NS	NS

**lubeluzole**, time point +180		**ANOVA**: k-1 = 5, N-k = 24, F=5.3722, P<0.002			
**mg/kg**	**0 (vehicle)**	**0.03**	**0.1**	**0.3**	**1**
**0.03**	NS				
**0.1**	NS	NS			
**0.3**	NS	P<0.05	NS		
**1**	P<0.001	P<0.001	P<0.01	NS	
**3**	P<0.01	P<0.01	NS	NS	NS

**riluzole**, time point +30		**ANOVA**: k-1 = 4, N-k = 19, F=35.2432, P<0.0001		
**mg/kg**	**0 (vehicle)**	**0.03**	**0.1**	**0.3**
**0.03**	NS			
**0.1**	P<0.001	P<0.01		
**0.3**	P<0.0001	P<0.0001	P<0.01	
**3**	P<0.0001	P<0.0001	P<0.001	NS

**riluzole**, time point +60		**ANOVA**: k-1 = 4, N-k = 19, F=15.4652, P<0.0001		
**mg/kg**	**0 (vehicle)**	**0.03**	**0.1**	**0.3**
**0.03**	NS			
**0.1**	P<0.05	P<0.01		
**0.3**	P<0.0001	P<0.0001	NS	
**3**	P<0.0001	P<0.0001	P<0.05	NS

**propafenone**, time point +30		**ANOVA**: k-1 = 4, N-k = 19, F=29.3967, P<0.0001		
**mg/kg**	**0 (vehicle)**	**1**	**5**	**20**
**1**	NS			
**5**	P<0.0001	P<0.001		
**20**	P<0.0001	P<0.001	NS	
**40**	P<0.0001	P<0.0001	NS	NS
